# An Ethyl Acetate Extract of *Eryngium carlinae* Inflorescences Attenuates Oxidative Stress and Inflammation in the Liver of Streptozotocin-Induced Diabetic Rats

**DOI:** 10.3390/antiox12061235

**Published:** 2023-06-07

**Authors:** Cristian M. Trejo-Hurtado, Cinthia I. Landa-Moreno, Jenaro Lemus-de la Cruz, Donovan J. Peña-Montes, Rocío Montoya-Pérez, Rafael Salgado-Garciglia, Salvador Manzo-Avalos, Christian Cortés-Rojo, Juan Luis Monribot-Villanueva, José Antonio Guerrero-Analco, Alfredo Saavedra-Molina

**Affiliations:** 1 Instituto de Investigaciones Químico Biológicas, Universidad Michoacana de San Nicolás de Hidalgo, Morelia 58030, Mexico; 1920749g@umich.mx (C.M.T.-H.); 1419561g@umich.mx (C.I.L.-M.); 1315680c@umich.mx (J.L.-d.l.C.); 0618853j@umich.mx (D.J.P.-M.); rmontoya@umich.mx (R.M.-P.); rsalgado@umich.mx (R.S.-G.); smanzo@umich.mx (S.M.-A.); christian.cortes@umich.mx (C.C.-R.); 2 Red de Estudios Moleculares Avanzados, Clúster BioMimic®, Instituto de Ecología, A.C., Xalapa 91073, Mexico; juan.monribot@inecol.mx (J.L.M.-V.); joseantonio.guerrero@inecol.mx (J.A.G.-A.)

**Keywords:** antioxidants, anti-inflammatory activity, diabetes mellitus, *Eryngium carlinae*, phenolic compounds, rosmarinic acid

## Abstract

Secondary metabolites such as flavonoids are promising in the treatment of non-alcoholic fatty liver disease (NAFLD), which is one of the complications of diabetes due to oxidative stress and inflammation. Some plants, such as *Eryngium carlinae*, have been investigated regarding their medicinal properties in in vitro and in vivo assays, showing favorable results for the treatment of various diseases such as diabetes and obesity. The present study examined the antioxidant and anti-inflammatory effects of the phenolic compounds present in an ethyl acetate extract of the inflorescences of *Eryngium carlinae* on liver homogenates and mitochondria from streptozotocin (STZ)-induced diabetic rats. Phenolic compounds were identified and quantified by UHPLC-MS. In vitro assays were carried out to discover the antioxidant potential of the extract. Male Wistar rats were administered with a single intraperitoneal injection of STZ (45 mg/kg) and were given the ethyl acetate extract at a level of 30 mg/kg for 60 days. Phytochemical assays showed that the major constituents of the extract were flavonoids; in addition, the in vitro antioxidant activity was dose dependent with IC50 = 57.97 mg/mL and IC50 = 30.90 mg/mL in the DPPH and FRAP assays, respectively. Moreover, the oral administration of the ethyl acetate extract improved the effects of NAFLD, decreasing serum and liver triacylglycerides (TG) levels and oxidative stress markers and increasing the activity of the antioxidant enzymes. Likewise, it attenuated liver damage by decreasing the expression of NF-κB and iNOS, which lead to inflammation and liver damage. We hypothesize that solvent polarity and consequently chemical composition of the ethyl acetate extract of *E. carlinae*, exert the beneficial effects due to phenolic compounds. These results suggest that the phenolic compounds of the ethyl acetate extract of *E. carlinae* have antioxidant, anti-inflammatory, hypolipidemic, and hepatoprotective activity.

## 1. Introduction

Diabetes mellitus is a metabolic disorder characterized by increased blood glucose or hyperglycemia [1]. Hyperglycemia is one of the causes of the development of various complications such as non-alcoholic fatty liver disease (NAFLD), in which there is an accumulation of lipids in the liver greater than 5–10%; in some cases, there is evidence of inflammation progressing to non-alcoholic steatohepatitis (NASH). This accumulation of lipids derived from an imbalance in their acquisition and disposition leads to metabolic alterations that result in an increase in the state of oxidative stress and mitochondrial dysfunction, which, in turn, leads to an inflammatory response and damage to and the death of hepatocytes [2,3].

The pharmacological treatment of NAFLD in patients with diabetes mellitus (DM) is based on reducing lipid accumulation and stopping the progression of inflammation and fibrosis. In spite of being considered promising for showing potential effects during in vitro and in vivo trials, few positive results have been reported in clinical trials confirming its effectiveness because of the multiple pathways implicated in the etiology of NAFLD [4,5]. However, several reports have shown that the use of extracts or bioactive compounds isolated from medicinal plants used in traditional medicine can prevent or control NAFLD [6,7].

Several species of *Eryngium* L. have been used in traditional medicine, such as *Eryngium carlinae*. It is commonly known as “frog herb”. In traditional medicine in Mexico, infusions of *E. carlinae* have been used to treat different conditions such as coughing, indigestion, prostate diseases, lipid disorders and diabetes [8,9]. Moreover, different biological activities of the plant have been demonstrated through in vitro and in vivo trials, such as the hypolipidemic activity of an ethanolic extract of the aerial part of *E. carlinae* (30 mg/kg and 100 mg/kg) in streptozotocin (STZ)-induced diabetic rats and hypercholesterolemic mice [10,11], the hypoglycemic and antioxidant activity of an aqueous extract of inflorescences of *E. carlinae* (0.6 g/day) in obese rats [12], the antioxidant activity of a hexanic extract of the inflorescences of *E. carlinae* (10 mg/mL in vitro and 30 mg/kg in vivo) in STZ-induced diabetic rats [13,14], and the anti-inflammatory activity of an ethanolic extract of *E. carlinae* stems and leaves in an acute inflammation model [15]. These biological activities are attributed to the content of secondary metabolites extracted according to polarity, which include: sesquiterpenes, terpenes, phytosterols, saponins, and flavonoids. Several flavonoids have been reported to exhibit favorable biological activities for the treatment of NAFLD through their effects on metabolism and anti-inflammatory potential [16]. The mechanism by which they act is through different pathways, which include decreasing the expression of fatty acid transporters, inhibiting lipogenesis, increasing β-fatty acid oxidation, enhancing antioxidant defense, and suppressing NF-κB pathway activation [17,18].

The aim of the present study is to determine the antioxidant and anti-inflammatory effects of the major secondary metabolites present in an ethyl acetate extract of the inflorescences of *Eryngium carlinae* on the livers of STZ-induced diabetic rats.

## 2. Materials and Methods

### 2.1. Plant Material

Inflorescences of *E. carlinae* were collected in September and November 2019 from Morelia, Michoacán, México. The plant was identified by Miguel Angel Bello-González PhD (Faculty of Agrobiology, Universidad Michoacana de San Nicolás de Hidalgo) genus and species preserving. A voucher specimen was deposited at the Biology Faculty Herbarium of the Universidad Michoacana de San Nicolás de Hidalgo (no. 15214). The inflorescences were dried, powdered, extracted with ethyl acetate (1:10 *w/v*) and kept at 4 °C for 10 days. The extract was dried under a vacuum using a rotary evaporator, suspended in mineral oil (150 mg/mL), and stored in the dark at 4 °C.

### 2.2. Phytochemical Constituents of the Ethyl Acetate Extract

#### 2.2.1. Total Flavonoid Content (TFC)

The TFC of the extract was measured according to a colorimetric assay [19]. For this, 10 µL of the ethyl acetate extract (1 mg/mL) was added to a 490 µL of methanol for 1 min, followed by 1000 µL of methanol, 100 µL of aluminum chloride (10%), and 100 µL of potassium acetate (1 M). The mixtures were incubated at room temperature for 30 min. The absorbance of the mixture was determined at 415 nm. A calibration curve was prepared from the different concentrations of quercetin (y = 0.016x − 0.0114). The TFC was reported as mg of quercetin equivalents per mL of the extract (mg QE/mL).

#### 2.2.2. Total Terpenoid Content (TTC)

The TTC in the ethyl acetate extract was determined by the method described by Ghorai et al. (2012) [20]. For this, 250 µL of the extract (10 mg/mL) was mixed with 2.5 mL of chloroform. The mixture was then vortexed thoroughly for 3 min and left to stand for 10 min on ice. Subsequently, 100 μL of concentrated sulfuric acid was poured into the mixture, followed by incubation at room temperature for 2 h in the dark. A reddish-brown precipitate was carefully taken and mixed with 900 µL of methanol. The absorbance was measured at 538 and the TTC was reported as mg of linalool equivalents per mL of the extract (mg LE/mL). A calibration curve was prepared from different concentrations of linalool (y = 0.0261x + 0.0172).

#### 2.2.3. Identification and Quantification of Phenolic Compounds of the Ethyl Acetate Extract

The identification and quantification of phenolic compounds from the ethyl acetate extract of *E. carlinae* was carried out as it was previously reported by Juárez-Trujillo et al. (2018) [21] and Monribot et al. (2019) [22]. The analysis was performed on a 1290 infinity Agilent ultra-high performance liquid chromatography system coupled to a 6460 Agilent triple quadrupole mass spectrometer (UHPLC-MS) with an Agilent, Eclipse Plus C18, 2.1 × 50 mm, 1.8 Microns column. The mobile phase consisted of water with 0.1% of formic acid (A) and acetonitrile with 0.1% formic acid (B) at a flow rate of 0.3 mL/min. The column temperature was 40 °C and 2 µL of sample was injected. The mass spectrometry conditions were set as follows: gas temperature, 300 °C; gas flow, 5 L/min; nebulizer pressure, 45 psi; sheath gas temperature, 250 °C; sheath gas flow, 11 L/min; capillary voltage (positive and negative), 3500 V and nozzle voltage (positive and negative), 500 V.

### 2.3. In Vitro Antioxidant Activity of the Ethyl Acetate Extract

#### DPPH Radical Scavenging Activity

The radical scavenging assay was performed using 1,1 diphenyl-2-picrylhydrazyl (DPPH) with some modifications [23,24]. Briefly, four different concentrations of the plant extract were mixed with 1 mL of ethyl acetate. Next, 1 mL of 0.2 mM DPPH in ethyl acetate was added. The mixture was shaken and left to stand in the dark at room temperature for 30 min. The absorbances of the mixtures were measured at 517 nm. The radical scavenging activity of the extract was reported as the half maximal inhibitory concentration (IC50).

### 2.4. In Vivo Biological Activity of the Ethyl Acetate Extract

#### 2.4.1. Animals

In the present study, 32 male Wistar rats weighing 300–350 g were housed in a controlled environment with free access to a standard diet and water, and a normal 12 h dark/light cycle. The animals were kept and used according to the recommendations of the Mexican Federal Regulations for the Use and Care of Animals (NOM-062-ZOO-1999, Ministry of Agriculture, México). Diabetes was induced after a 14 h fasting period with an intraperitoneal administration of streptozotocin (STZ, 45 mg/kg of body weight) dissolved in a citrate buffer (0.1 M, pH 4.5). Normoglycemic groups were injected with a citrate buffer (the vehicle). After 5 days of STZ administration, glucose levels were determined with a commercial glucometer (Accu-Chek Performa, Roche Diagnostics GmbH, Mannheim, Germany) to confirm diabetes. Rats exhibiting blood glucose levels >300 mg/dL were considered for the study.

The rats were divided randomly into four groups (n = 8) comprising a normoglycemic control (vehicle and mineral oil), a diabetic control (STZ and mineral oil), a normoglycemic + EC group (vehicle and *E. carlinae* extract at a dose of 30 mg/kg), and a diabetic + EC group (STZ and *E. carlinae* extract at a dose of 30 mg/kg). The rats were treated with either mineral oil or the extract 15 days after citrate/STZ administration for 60 days. During this period, blood glucose concentrations was determined, and weight was measured once a week.

At the end of the 60 day-treatment, the rats were fasted overnight and euthanized. Blood samples were collected, and the serum was separated by centrifugation at 5000 rpm for 10 min. Serum was used for determining glucose, triacylglycerides (TG), and the liver enzymes alanine aminotransferase (ALT), aspartate aminotransferase (AST), and alkaline phosphatase (ALP).

The liver was excised and placed in ice-cold PBS (pH 7.4). Homogenate was prepared and mitochondria were isolated by differential centrifugation of liver homogenates as described elsewhere [25]. Samples of the homogenate and mitochondria were stored at −70 °C until use. Protein concentrations were assessed before each assay by the Biuret and Lowry (for immunoblotting) methods.

#### 2.4.2. Liver Triacylglycerides Content

Liver triacylglycerides from homogenate samples were obtained by the method of Aguilera-Méndez and Fernández-Mejía with some modifications [26]. Briefly, 20 µg/µL of protein was resuspended in 1 mL of a solution containing 5% Triton-X100 in a PBS buffer. Samples were subjected to water bath warming at 60 °C for 5 min and cooled down slowly at room temperature. The samples were centrifuged at 13,300 rpm for 15 min. Triacylglycerides concentrations were determined from the supernatant with the reagent kit Spin react (BSIS49-E, Sant Esteve De Bas, Spain).

#### 2.4.3. Oxidative Stress Markers

ROS generation was determined by evaluating the oxidation of the fluorescence probe 2′,7′-dichlorodihydrofluorescein diacetate (H_2_DCFDA) [27]. In brief, 0.1 mg/mL of freeze-thawed mitochondrial protein was resuspended in a buffer containing 10 mM HEPES, 100 mM KCl, 3 mM MgCl_2_, and 3 mM KH_2_PO_4_ (pH 7.4) and incubated with 12.5 µM H_2_DCFDA for 15 min in an ice bath with shaking. Next, 10 µL of rotenone was added to the mixture and basal fluorescence was recorded for 1 min, then 10 mM of succinate was added. Changes in the fluorescence were recorded for 19 min at excitation/emission wavelengths of 491/518 nm.

Lipid peroxidation was assessed in liver mitochondria by measuring the levels of thiobarbituric acid reactive substances (TBARS), according to the protocol modified by Peña-Montes from Buege and Aust [28]. Briefly, 0.5 mg/mL of protein was washed in PBS (pH 7.4) through two cycles of decantation and centrifugation at 10,000 rpm for 10 min at 4 °C. The precipitate was then resuspended in 300 µL of PBS. Next, 0.5 mg/mL of washed mitochondria was mixed with a reagent solution (0.375% thiobarbituric acid (TBA), 15% trichloroacetic acid and 0.25 M HCl) and incubated for 30 min in a boiling water bath. After cooling, the flocculent precipitate was centrifuged at 7500 rpm for 5 min. The absorbance was measured at 532 nm. Lipid peroxidation was calculated based on the absorbance, with 156 mM^−1^cm^−1^ as the molar extinction coefficient.

#### 2.4.4. Antioxidant Enzyme Activities

Catalase and superoxide dismutase (MnSOD) were assessed in the liver homogenate and mitochondria, respectively. Catalase activity was measured by the conversion of hydrogen peroxide to oxygen according to a method reported previously [29]. In brief, 0.1 mg/mL of protein was resuspended in 0.1 M phosphate buffer and 5 mM EDTA (pH 7.6) at 25 °C and monitored for 1 min. Next, 5 mM H_2_O_2_ was added to the chamber, and the conversion of hydrogen peroxide to oxygen was measured with an oxygen electrode for 3 min. MnSOD activity was determined by a commercial kit (Sigma-Aldrich, St. Louis, MO, USA) 19160 SOD determination kit) following the manufacturer’s instructions.

#### 2.4.5. Mitochondrial Respiratory Chain Complex Activities

To evaluate the activities of the respiratory chain complexes, mitochondria were permeabilized by freeze-thawing. Complex I activity was assessed by a method previously described, with some modifications [27]. For this, 0.05 mg/mL of protein was resuspended and incubated in deionized water for 2 min. Next, a 250 mM phosphate buffer, 10 mg/mL BSA, 0.5 mM antimycin A and 0.2 mM KCN were added, mixed, and incubated for 7 min at room temperature. This was then mixed with 10 mM K_3_[Fe(CN)_6_] before recording the basal fluorescence for 1 min at excitation/emission wavelengths of 352/464 nm. Next, 10 mM β-NADH was added, and changes in fluorescence were monitored for 2 min. The same procedure was performed for rotenone-insensitive Complex I activity by adding 2 mM rotenone. The specific activity was calculated using a standard curve for β- NADH.

The activities of Complexes II and II + III were determined as described previously [30]. For Complex II, 0.1 mg/mL of solubilized mitochondria was resuspended in a 250 mM phosphate buffer, 10 mg/mL BSA, 0.5 M succinate, 2 mM rotenone, 0.5 mM antimycin A, and 0.2 mM KCN. The mixture was incubated for 3 min at room temperature, and then 80 µM DCIP was added, and the basal absorbance was monitored for 2.5 min at 600 nm. Next, 100 mM TTFA was added, and the changes in absorbance were monitored for 2.5 min. The specific activity was calculated based on the absorbance, with 21 mM^−1^ cm^−1^ as the molar extinction coefficient. Whereas, for the activity of Complex II + III, 0.1 mg/mL of the solubilized mitochondria was resuspended in a 250 mM phosphate buffer, 20 mM EDTA, 10 mg/mL BSA, 0.5 M succinate, 2 mM rotenone, and 0.2 mM KCN. The mixture was incubated for 3 min at room temperature, then 250 µg oxidized cytochrome c was added to the mixture, and the basal absorbance was recorded for 1.5 min at 550 nm. Finally, 0.5 mM antimycin A was added, and the changes in absorbance were measured for 3 min. The specific activity was calculated based on the absorbance, with 19.1 mM^−1^ cm^−1^ as the molar extinction coefficient.

Complex IV activity was evaluated by the protocol described by Peña-Montes et al. (2020) [31]. For this, 0.1 mg/mL of solubilized mitochondria was resuspended in a 250 mM phosphate buffer, 10 mg/mL BSA, 2 mM rotenone, 100 mM TTFA, and 0.5 mM antimycin A. The mixture was incubated for 3 min at room temperature. Next, 125 µg of reduced cytochrome c was added, and the basal absorbance was recorded for 24 s at 550 nm. Next, 0.2 mM KCN was added, and the changes in absorbance were monitored for 2 min. The specific activity was calculated based on the absorbance with 19.1 mM^−1^ cm^−1^ as the molar extinction coefficient.

#### 2.4.6. Expression of Inflammatory Markers

The expression levels of transcription factor NF-κΒ and iNOS protein in the liver homogenate were measured by immunoblotting. Briefly, 50 µg of protein was resolved on 10% SDS polyacrylamide gels, and then transferred to polyvinylidene fluoride membranes and blocked with 5% (*w/v*) nonfat milk in TBS-T (10 mM Tris/HCl, 150 mM NaCl (pH 7.6) and 0.05% TWEEN 20) for 1 h at 20 °C. Next, the membranes were incubated overnight at 4 °C on a shaker with primary anti- NF-κΒ (1:400; ab-7971, Abcam, Cambridge, UK), anti-NOS2 (1:200; sc-7271, Santa Cruz, CA, USA), or anti-β-tubulin (1:200; sc-55529, Santa Cruz, CA, USA). The samples were incubated for 2 h or overnight at 4 °C on a shaker with HRP-conjugated goat anti-rabbit IgG (1:4000; sc-2004, Santa Cruz, CA, USA) or m-IgGκ BP (1:1000; sc-516102, Santa Cruz, CA, USA). Images were acquired using chemiluminescence and a ChemiDoc XRS+ imaging system (Bio-Rad, Hercules, CA, USA), and were analyzed with ImageLab 6.0.1. (Bio-Rad, Hercules, CA, USA). The relative protein levels were calculated based on β-tubulin expression as a loading control.

### 2.5. Statistical Analysis

The in vitro results are reported as means ± standard deviation, while the in vivo results are expressed as means ± standard error. The significance of differences between the means were assessed using one-way ANOVA, followed by Tukey’s multiple comparison test. A *p* value of < 0.05 was considered statistically significant.

## 3. Results

### 3.1. Determination of the Phytochemical Constituents of the Ethyl Acetate Extract

The results for the TFC and TTC of the ethyl acetate extract of E. carlinae inflorescences are shown in Table 1. The TFC and TTC of the extract were 4.097 ± 0.0715 mg QE/mL and 0.8105 ± 0.0224 mg LE/mL, respectively. However, it was found that the ethyl acetate extract had high values of total flavonoid content.

The phenolic compounds of the ethyl acetate extract of E. carlinae inflorescences were identified and quantified by UHPLC-MS as shown in Table 2. The most abundant compound was rosmarinic acid (3473.79 ± 146.18 µg/g), followed by chlorogenic acid (64.92 ± 1.24 µg/g) and kaempferol-3-O-glucoside (50.42 ± 1.72 µg/g).

### 3.2. Determination of the In Vitro Antioxidant Activity of the Ethyl Acetate Extract

**Figure 1:**
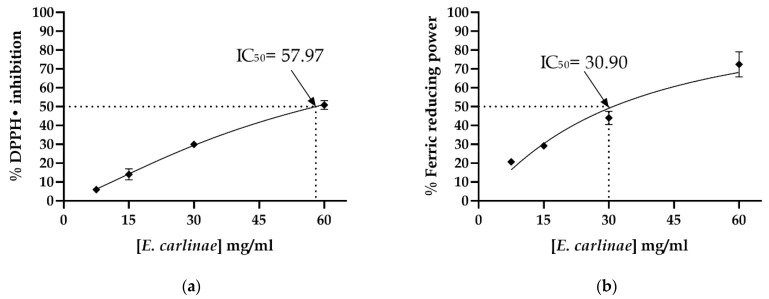
In vitro antioxidant activity of the ethyl acetate extract of the inflorescences of *E. carlinae*: (**a**) DPPH radical-scavenging activity; (**b**) FRAP assay. Results are shown as the mean ± SD of three independent experiments.

To determine the in vitro antioxidant activity of the *E. carlinae* extract, DPPH and FRAP assays were applied, which explored the hydrogen atom and single electron mechanisms. As shown in Figure 1, the extract had radical-scavenging and ferric-reducing ability in a dose-dependent manner, with IC50 = 57.97 mg/mL and IC50 = 30.90 mg/mL respectively, thus displaying higher in vitro antioxidant activity through the single electron mechanism.

### 3.3. Effect of the Ethyl Acetate Extract on Biochemical Parameters and Body Weight Gain In Vivo

At the end of the 60 days of treatment, the glucose levels of diabetic rats were 4.6-fold higher than those of normoglycemic rats. Administration of the ethyl acetate extract did not have any effect on this parameter in both groups compared with the respective controls (Table 3). Besides glucose, the triacylglycerides levels of diabetic rats were 1.5-fold higher than those in normoglycemic rats. However, this increase was normalized with administration of the extract in diabetic rats. Regarding the body weight gain of the different groups, diabetic rats exhibited an 18.7% weight loss at the end of the treatment, whereas normoglycemic rats had a weight gain of 20.4%. This weigh loss was attenuated by treatment with the extract in diabetic animals, as their weight loss was 1.4%.

### 3.4. Influence of the Ethyl Acetate Extract on Liver Enzymes and Triacylglycerides Content

Serum levels of the liver enzymes AST, ALT, and ALP were measured to determine liver damage (Table 4). The increase in the levels of these enzymes in the diabetic group was 1.8, 3, and 14.3-fold higher than those in the normoglycemic group, which was indicative of liver damage. However, this increase was attenuated by about 26.5%, 38.6%, and 46.9%, respectively, by daily administration of the extract. Moreover, the administration of the extract to the normoglycemic group did not significantly alter the levels of these enzymes. The influence of the ethyl acetate extract on the amount of TG in the liver was determined as well. The TG content in diabetic rats increased by 2.4-fold compared with normoglycemic rats, while the extract could keep the TG content at levels statistically like those of the normoglycemic rats.

### 3.5. Effect of the Ethyl Acetate Extract on Oxidative Stress Markers

**Figure 2:**
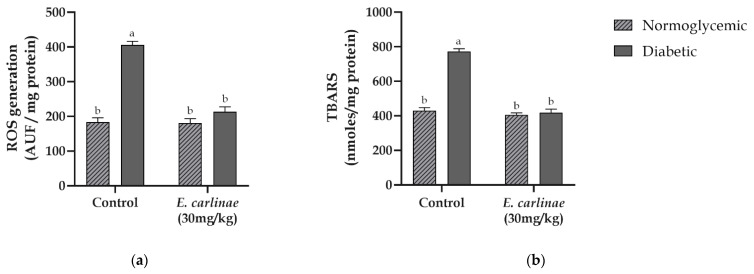
Effects of the ethyl acetate extract of the inflorescences of *E. carlinae* on oxidative stress markers: (**a**) mitochondrial ROS generation; (**b**) lipid peroxidation. Values represent the mean ± SE, n = 8. Different letters indicate significant differences among treatments (*p* < 0.05), based on one-way ANOVA and Tukey’s post-hoc test.

Mitochondrial ROS generation and lipid peroxidation were assessed in order to determine the degree of oxidative stress in the liver in response to hyperglycemia and administration of the extract. As shown in Figure 2, ROS in diabetic rats were 2.2-fold higher than those in normoglycemic rats. Similar behavior was observed when lipid peroxidation was evaluated in control rats (1.8-fold higher in diabetic rats than in normoglycemic rats). In contrast, administration of the extract inhibited ROS production and prevented mitochondrial membrane oxidative damage in diabetic rats at levels similar to those of the normoglycemic groups.

### 3.6. Influence of the Ethyl Acetate Extract on Liver Antioxidant Enzyme Activity

**Figure 3:**
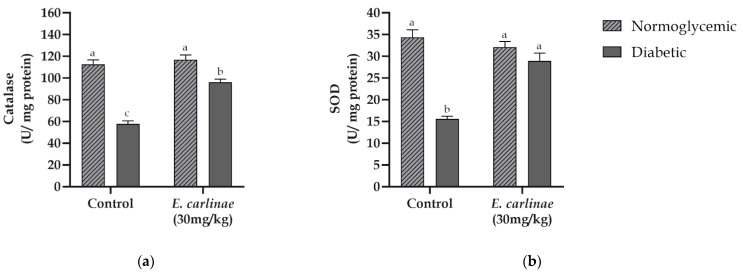
Influence of the ethyl acetate extract of the inflorescences of *E. carlinae* on liver antioxidant enzyme activity: (**a**) catalase activity; (**b**) mitochondrial SOD activity. Values represent the mean ± SE, n = 8. Different letters indicate significant differences among treatments (*p* < 0.05), based on one-way ANOVA and Tukey’s post-hoc test.

To determine the in vivo antioxidant activity of the extract, its effects on the activity of two important antioxidant enzymes in the detoxification of ROS, namely, catalase in the homogenate and SOD in the liver mitochondria, were measured (Figure 3). The catalase activity levels of the diabetic group were significantly lower than those from the normoglycemic group (0.5-fold lower). Moreover, the SOD activity of diabetic rats showed a significant decrease of about 0.45-fold compared with the activity of this enzyme in normoglycemic rats. This phenomenon was restored when the extract was administered to the diabetic group, which showed a significant increase of 65% and 86% compared with the untreated diabetic group, respectively, for catalase and MnSOD. 3.7.

### 3.7. Effect of the Ethyl Acetate Extract on Mitochondrial Complex Activities

**Figure 4:**
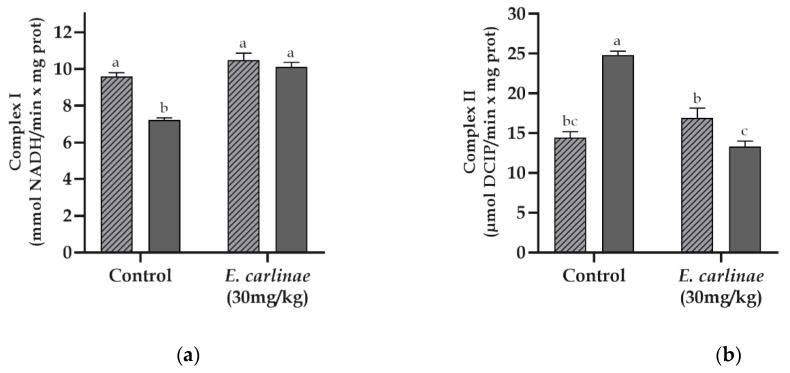

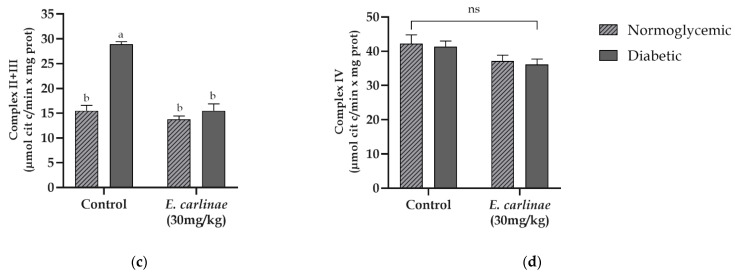
Effect of the ethyl acetate extract of the inflorescences of *E. carlinae* on mitochondrial complex activities: (**a**) Complex I; (**b**) Complex II; (**c**) Complex II + III; (**d**) Complex IV. Values represent the mean ± SE, n = 8. Different letters indicate significant differences among treatments (*p* < 0.05), based on one-way ANOVA and Tukey’s post-hoc test.

The activity of the complexes from the electron transport chain was assessed in order to determine the degree of dysfunction and the effect of the ethyl acetate on its functionality. The activity of Complex I in diabetic rats showed a significant decrease of 0.8-fold lower compared with the normoglycemic group (Figure 4a), while the activity of Complexes II and II + III (Figure 4b,c) displayed an increase in activity that was 1.7 and 1.9-fold higher in diabetic rats compared with the rats from the normoglycemic group, respectively. However, the dysfunction of Complexes I, II and II + II was prevented in diabetic rats administered the extract. In contrast, Complex IV activity did not show any significant changes among the groups (Figure 4d).

### 3.8. Effect of the Ethyl Acetate Extract on the Expression of Inflammatory Markers

**Figure 5:**
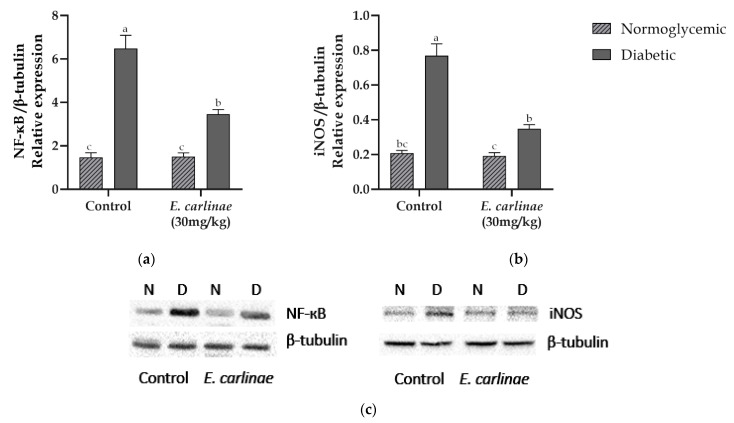
Effect of the ethyl acetate extract of the inflorescences of *E. carlinae* on the expression of inflammatory markers: (**a**) densitometric analysis of NF-κB expression; (**b**) densitometric analysis of iNOS expression; (**c**) representative immunodetection images. Values represent the mean ± SE, n = 3. Different letters indicate significant differences among treatments (*p* < 0.05), based on one-way ANOVA and Tukey’s post hoc test.

Protein expression levels of the inflammatory markers NF-κΒ transcription factor and iNOS were measured to determine the anti-inflammatory activity of the ethyl acetate extract (Figure 5). The expression of both proteins was significantly upregulated in diabetic rats by 4.4- and 3.7-fold compared with the normoglycemic group, respectively, for NF-κΒ transcription factor and iNOS. NF-κB and iNOS were significantly downregulated by treatment with the ethyl acetate extract by 0.5- and 0.45-fold compared with the diabetic group.

## 4. Discussion

In the present study, it was demonstrated that one of main groups of secondary metabolites extracted from the inflorescences with ethyl acetate was flavonoids (Table 1), a subgroup of phenolic compounds reported to be found in this genus besides saponins and essential oils [32]. This result is consistent with some reports in the literature which demonstrated a high content of total flavonoids in extracts of ethyl acetate from the inflorescences of *E. kotschyi* and the aerial parts of *E. campestre*, *E. amethystinum,* and *E. palmatum* with respect to other solvents [33,34].

However, rosmarinic acid was the phenolic compound found in the highest concentration (Table 2), which is in agreement with the high concentration of this compound in an *E. kotschyi* ethyl acetate sub-extract obtained by Paşayeva et al. [33]; and by Kikowska et al. who demonstrate the presence of high concentrations of rosmarinic acid in root and shoot extracts of three Eryngium species [35]. Chlorogenic acid and kaempferol-3-O-glucoside were found in much lower concentrations than rosmarinic acid, but relatively higher than for other phenolic compounds.

The antioxidant activity of the ethyl acetate extract of *E. carlinae* inflorescences was evaluated through DPPH and reducing power assays, which are based on the inactivation of an oxidant molecule (Figure 1). It was demonstrated that the ethyl acetate extract’s antioxidant activity was related to the arrangement of the functional groups of the phenolic compounds, mainly the hydroxyl groups that carry out the transfer of electrons or the donation of H^+^ atoms for radical stabilization or chelation of metal ions [36,37]. Compared with the DDPH anti-radical activity of the ethyl acetate extract from *E. carlinae* inflorescences and the aerial parts of *E. maritimum* (IC_50_ = 31.19 mg/mL), our extract showed lower antioxidant activity [38]. However, the reducing power of our extract was 30.90 mg/mL, a similar concentration to that reported for a hexanic extract of inflorescences of *E. carlinae*, which was statistically greater than or equal to that of the standard antioxidant used [14]. However, these differences may be due to the main content of secondary metabolites, in this case phenolic compounds, which varies according to the environmental conditions and among species, reducing the effect of the antioxidant potential of the *E. carlinae* extract compared with other plants [39].

During diabetes, besides hyperglycemia, a characteristic symptom of the disease is the loss of weight, especially in Type 1 diabetes. This is because lipids and proteins are more prone to be metabolized than carbohydrates, and dyslipidemia, which, due to insulin deficiency or resistance, promotes an increase in TG accumulation in the serum and liver [40,41]. The results from this study showed that the ethyl acetate extract was not able to lower blood glucose levels, as was reported for an ethanolic extract of the aerial parts of *E. carlinae* [10,42]. Nevertheless, the administration of the ethyl acetate extract promoted a decrease in weight loss and serum TG content. This hypolipidemic activity is in accordance with Noriega-Cisneros et al. [10], who demonstrated that the presence of stigmasterol promoted a significant decrease in serum TG content through the modulation of de novo lipogenesis and lipid absorption in the diabetic group treated with 30 mg/mL of an ethanolic extract of *E. carlinae* [40]. A decrease in hepatic TG content (Table 4) was also shown when an ethyl acetate extract was administered, as reported by Murillo-Villicaña et al. [43,44] through the administration of an ethyl acetate extract of *Justicia spicigera* in STZ-induced diabetic rats. This hypolipidemic effect could be related to the high content of rosmarinic acid, promoting fatty acid β-oxidation via AMPK and by inhibiting fatty acid synthesis, leading to a decrease in hepatic TG content [45,46].

There is clinical and experimental evidence of the use of different methods for NAFLD/NASH diagnosis. The assessment of liver enzymes such as ALT, AST, and ALP can be taken into account for an initial diagnosis of the disease [47]. In this study, the increased levels of the enzymes of the diabetic group (Table 4) were related to necrosis and fibrosis events that occur during NAFLD progression [48], while the decrease in the levels of these enzymes produced by the extract in the diabetic group could be related to the content of flavanones such as naringin, which has shown a hepatoprotective effect by decreasing ATL and AST levels and mitigating morphological changes [49,50]. However, either liver biopsies or histopathological analyses need to be carried out to determine the presence, severity, and the stage of NAFLD [51].

Oxidative stress derived from lipid accumulation has been shown to dysregulate liver signaling and metabolism, leading to the development of liver disease [3,52]. Therefore, the activity of the ethyl acetate extract and their effects on the effects triggered by lipid accumulation were determined.

One of the effects of lipid accumulation is an increase in ROS production by the mitochondria in an attempt to decrease the lipid load through β-oxidation [3]. As shown in Figure 2a, there was an increase in ROS production in the diabetic group, while this effect was mitigated by administration of the extract. Moreover, this increase in ROS led to mitochondrial membrane lipid peroxidation [53]. As expected from the results of ROS generation, the lipid peroxidation levels of diabetic rats treated with the ethyl acetate extract showed a decrease in lipid peroxidation of about the half of that in the diabetic group (Figure 2b). This prevention of oxidative damage could be related to the antioxidant activity of flavonoids and phenolic acids in the extract, especially rosmarinic acid which is able to scavenge ROS by the enhancement of antioxidant defense enzymes [54]; and inhibit lipid peroxidation through its association within the lipid membrane as previously reported by Fadel et al. [55].

During lipid overload, oxidation is mediated by the cytochromes and peroxisomes, contributing to the production of ROS, leading to an imbalance between the antioxidant system and ROS [3]. In this study, the diabetic group showed a decrease in catalase and MnSOD activity (Figure 3), which is consistent with other studies that have reported that during hyperglycemia or steatosis, both enzymes showed a decrease in their activities [53,56]. However, administration of the extract significantly increased the enzymatic activity in diabetic rats, so it is possible that rosmarinic acid enhances the antioxidant system through Nrf2 signaling pathway and upregulating the downstream antioxidant enzymes [57].

Additionally, the byproducts of lipid peroxidation cause alterations in the mitochondrial membrane, contributing to dysfunction, an important feature of NAFLD, which leads to cell damage [3]. These alterations include cardiolipin oxidation, which is important for the structure and function of Complexes I and III [58], and related events in Complex I inhibition in the diabetic group (Figure 4a). They are related to the increase in lipid peroxidation in this group (Figure 2b), in which cardiolipin may be involved. Moreover, this inhibition could be related to the increase in the activity of Complex II in the diabetic group (Figure 4b) in a compensatory way to reduce the reducing equivalents of fatty acid oxidation by TG accumulation (Table 4), which is in agreement with the results obtained by Ortiz-Avila et al. in STZ-induced diabetic rats [27]. Regarding the increase in Complex III activity (Figure 4c), our results were not in accordance with those of other reports in which its activity was inhibited due to cardiolipin oxidation; however, our results could be at a stage prior to the inhibition reported by Moreira et al. [59]. Furthermore, its activity is related to the increase in ROS generation in diabetic rats, as shown in Figure 2a, through a highly reduced Q pool during the Q cycle [60]. However, administration of the ethyl acetate extracts restored Complex I activity, preventing cardiolipin oxidation, as it decreased lipid peroxidation in diabetic rats, and restored the activity of Complexes II and III, promoting a decrease in hepatic TG accumulation and restoring Complex I activity. In addition, the activity of Complex IV was not altered in any group, as reported in a diabetes and steatosis model, in which the activity of this complex did not show significant changes [27,53].

Lipid accumulation and the increase in oxidative stress induce an inflammatory response, another main characteristic of NAFLD. This inflammatory response in hepatocytes begins with NF-κΒ (p50:RelA heterodimer) transcription factor activation by release of its inhibitor Iκβ by phosphorylation and translocation to the nucleus, where it binds to DNA and promotes pro-inflammatory cytokines and inducible enzyme transcription [61]. In this study, an increase in the expression levels of NF-κΒ was observed in the diabetic group (Figure 5a), which was consistent with the increased expression of this transcription factor in diabetic rats reported by Tian et al. in type 2 diabetic rats [62], while the administration of the extract decreased this effect, as reported for rosmarinic acid administration in mouse model of nonalcoholic steatohepatitis [63]. However, this decrease in the diabetic group treated with the extract was not similar to the results from the normoglycemic groups, which could be due to the polarization process of the macrophages M1/M2, in which, during the transition modulated by flavonoids such as quercetin or apigenin, both phenotypes coexist and cytokines are still being secreted, so that the expression levels of this transcription factor remain high, as reported by Feng et al. in obese mice [64,65].

Once NF-κΒ has translocated to the nucleus, the transcription of inducible enzymes such as nitric oxide synthase (iNOS) is carried out. Under hyperglycemic conditions, this enzyme promotes inflammation and apoptosis in the liver, as this isoform, unlike the constitutive ones, can produce a large amount of nitric oxide (NO) from L-arginine. In addition, through different stimuli, such as ROS or the cytokines TNF-α and IL-1β, the expression of iNOS is increased not only in Kupffer cells (macrophages) but also in hepatocytes and hepatic stellate cells [66]. This is consistent with the results from the present study: Figure 5b shows that the diabetic group had an increase in the levels of iNOS expression due to the increase in ROS production (Figure 2a), and this is related to the increase in NF-κΒ expression levels (Figure 5a), which promoted the transcription of iNOS in response to ROS generation. Moreover, this result is related to the decrease in the antioxidant enzyme activity (Figure 3), because the generated NO leads to nitration and s-nitrosylation of these proteins. However, administration of the ethyl acetate extract to the diabetic group decreased iNOS expression levels, as reported by Lu et al., who found that the administration of rosmarinic acid decreased iNOS expression and NO production [57]. However, NO production and the expression levels of Nrf2 and proinflammatory cytokines such as TNF-α need to be studied further to support the information about the anti-inflammatory activity of the ethyl acetate extract of the inflorescences of *E. carlinae.*

## 5. Conclusions

**Figure 6:**
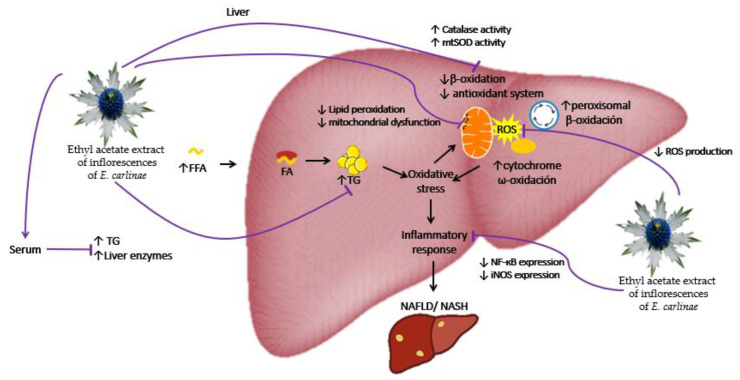
Hypothetical interaction model of the ethyl acetate extract of the inflorescences of *E. carlinae*. FA: fatty acids; FFA: free fatty acids; iNOS: inducible nitric oxide synthase; MnSOD: mitochondrial superoxide dismutase; NF-κΒ: nuclear factor κΒ; TG: triacylglycerides; ROS: reactive oxygen species.

The present results demonstrated that the phenolic compounds such as rosmarinic acid of the ethyl acetate extract of the *E. carlinae* inflorescences had dose-dependent antioxidant activity in vitro and in vivo at a dose of 30 mg/kg in STZ-induced diabetic rats. The antioxidant activity of the extract was demonstrated through a decrease in oxidative stress markers by inhibiting ROS production, decreasing lipid peroxidation, and restoring mitochondrial complex activity, as well as enhancing the antioxidant system by restoring the activity of the antioxidant enzymes catalase and MnSOD. In addition, the ethyl acetate extract had anti-inflammatory effects due to its ability to decrease NF-κΒ and iNOS expression. It also showed hypolipidemic activity in the serum and the liver of STZ-induced diabetic rats by decreasing TG levels, and it may also have a possible hepatoprotective effect, as observed through the decrease in liver enzymes in the serum. The proposed hypothetical interaction model of the ethyl acetate extract of the inflorescences of *E. carlinae* is shown in Figure 6.

## Figures and Tables

**Table 1:** Total flavonoid and terpenoid contents of the *Eryngium carlinae* extract.

TFC (mg QE/mL)	TTC (mg LE/mL)
4.097 ± 0.0715	0.8105 ± 0.0224

Values are expressed as mean ± SD of three independent experiments.

**Table 2:** Phenolic compounds of the ethyl acetate extract of *Eryngium carlinae* inflorescences.

Compound	Retention Time (min)	Concentration (µg/g of Dried Extract)	Compound	Retention Time (min)	Concentration (µg/g of Dried Extract)
Protocatechuic acid	2.5	23.53 ± 0.93	Salicylic acid	9.15	8.08 ± 0.42
4-Hydroxybenzoic acid	3.76	34.58 ± 1.23	Ellagic acid	9.98	3.34 ± 0.43
Vanillic acid	5.12	7.48 ± 0.20	Quercetin-3-D-Galactoside	10.26	4.03 ± 0.16
Chlorogenic acid	5.34	64.92 ± 1.24	p-Anisic acid	10.45	33.71 ± 0.95
Caffeic acid	5.38	26.11 ± 0.88	Kaempferol-3-O-glucoside	11.91	50.42 ± 1.72
Vanillin	6.52	22.76 ± 0.30	Rosmarinic acid	12.8	3473.79 ± 146.18
4-Coumaric acid	7.21	13.79 ± 0.35	Quercetin	15.18	3.96 ± 0.07
Umbelliferone	7.64	6.57 ± 0.23	Kaempferol	17.81	22.10 ± 0.45
Ferulic acid	8.6	4.77 ± 0.20	Nordihydroguaiaretic acid	22.72	5.51 ± 0.50

Values are expressed as mean ± SD of three independent experiments.

**Table 3:** Effects of the ethyl acetate extract of the inflorescences of *E. carlinae* on biochemical parameters and body weight gain at the end of 60 days of administration.

Group	Glucose (mg/dL)	TG (mg/dL)	Body Weight Gain (%)
Normoglycemic control	96.556 ± 4.231 ^b^	50.875 ± 3.861 ^bc^	20.425 ± 2.339 ^a^
Diabetic control	445.750 ± 20.385 ^a^	78.417 ± 3.355 ^a^	-18.707 ± 3.484 ^c^
Normoglycemic + EC	96.30 ± 3.491 ^b^	40.375 ± 2.983 ^c^	17.668 ± 1.439 ^a^
Diabetic + EC	440.988 ± 18.493 ^a^	58.125 ± 3.102 ^b^	1.400 ± 3.484 ^b^

Values represent the mean ± SE, n = 8. EC: ethyl acetate extract of *E. carlinae* at 30 mg/kg body weight; TG: triacylglycerides. Different letters indicate significant differences among treatments (*p* < 0.05), based on one-way ANOVA and Tukey’s post-hoc test.

**Table 4:** Effect of the ethyl acetate extract of the inflorescences of *E. carlinae* on liver enzymes and the liver triacylglycerides content at the end of 60 days of administration.

Group	AST (U/L)	ALT (U/L)	ALP (U/L)	Liver TG
Normoglycemic control	257.625 ± 25.230 ^c^	74.5 ± 6.039 ^c^	150.250 ± 19.118 ^c^	0.805 ± 0.068 ^b^
Diabetic control	462.571 ± 19.895 ^a^	223.571 ± 14.371 ^a^	2144.625 ± 91.351 ^a^	1.992 ± 0.022 ^a^
Normoglycemic + EC	201.125 ± 18.218 ^c^	55.75 ± 2.821 ^c^	136.5 ± 14.891 ^c^	0.860 ± 0.048 ^b^
Diabetic + EC	339.750 ± 17.045 ^b^	137.125 ± 7.988 ^b^	1137.143 ± 109.381 ^b^	0.951 ± 0.060 ^b^

Values represent the mean ± SE, n = 6. EC: ethyl acetate extract of *E. carlinae* at 30 mg/kg body weight; AST: aspartate aminotransferase; ALT: alanine aminotransferase; ALP: alkaline phosphatase; TG: triacylglycerides. Different letters indicate significant differences among treatments (*p* < 0.05), based on one-way ANOVA and Tukey’s post-hoc test.

## Data Availability

The data used to support the findings of this study are available from the corresponding author upon request.
